# Microalbuminuria as the Tip of Iceberg in Type 2 Diabetes Mellitus: Prevalence, Risk Factors, and Associated Diabetic Complications

**DOI:** 10.7759/cureus.43190

**Published:** 2023-08-09

**Authors:** Sohaib Asghar, Shoaib Asghar, Tayyab Mahmood, Syed Muhammad Hassan Bukhari, Muhammad Habib Mumtaz, Ali Rasheed

**Affiliations:** 1 Gastroenterology, Glan Clwyd Hospital, Betsi Cadwaladr University Health Board, Rhyl, GBR; 2 Internal Medicine, Sheikh Zayed Medical College and Hospital, Rahim Yar Khan, PAK; 3 Geriatric Medicine, King's College Hospital, NHS foundation Trust, London, GBR; 4 Medicine, King's College Hospital, NHS foundation Trust, London, GBR; 5 Colorectal Surgery, King's College Hospital, NHS foundation Trust, London, GBR

**Keywords:** diabetic peripheral neuropathy (dpn), diabetic retinopathy, dyslipidaemia, glycosylated haemoglobin (hba1c), hypertension, end stage renal disease (esrd), diabetic nephropathy (dn), albumin to creatinine ratio (acr), type 2 diabetes mellitus (t2dm), microalbuminuria (ma)

## Abstract

Background

Microalbuminuria (MA) is an important clinical marker for the early detection of kidney damage in patients with type 2 diabetes (T2DM). Urine albumin-to-creatinine ratio (ACR), also known as urine microalbumin, is a sign of diabetic nephropathy (DN), which is a prevalent complication of diabetes and can result in end-stage renal disease (ESRD) if not managed. The prevalence of MA in T2DM has been steadily increasing worldwide, making it a significant public health concern. The goal of this study was to estimate the prevalence of MA and its relationship to hypertension and other diabetic complications among people with T2DM.

Methodology

This descriptive cross-sectional study was conducted from February 5, 2022, to February 10, 2023, to analyse data from T2DM patients who visited the outpatient diabetic clinic of Sheikh Zayed Medical College and Hospital, Rahim Yar Khan, Pakistan. This study included a total of 640 patients, aged 35-60 years, who had been diagnosed with T2DM for at least five years and fulfilled the inclusion criteria.

Data on demographic and clinical characteristics, blood pressure (BP) measurements, and laboratory investigations were collected. MA was assessed based on the ACR in a spot urine sample of more than 30 mg/l. Blood pressure greater than 140/90 or already taking anti-hypertensives was taken to constitute hypertension. Factors associated with MA like hypertension, gender, mode of diabetes treatment, duration of diabetes, glycosylated haemoglobin (HbA1c), dyslipidemia, and other diabetic complications such as retinopathy and neuropathy were also recorded.

Results

The prevalence of MA in this study of T2DM patients study was 39.1%. The mean age of the participants with MA was 53.9 with a standard deviation (SD) of 6.1 years, and the mean duration of diabetes was 10.1 years (SD 6.2 years); 101 (33.4%) males (n=302) and 103 (30.5%) females (n=338) had MA.

There was a statistically significant correlation between MA > 30mg/d and hypertension (p = <0.001), diabetes duration since diagnosis (p=0.04), HbA1C level (p = <0.001), dyslipidemia (p=0.001), therapy type (p = <0.001), triglyceridemia (p = 0.03), history of diabetes retinopathy (p= <0.002), and peripheral neuropathy (p= <0.001). However, there was no statistically significant correlation between MA and age (p = 0.56), female gender (p = 0.08), low- and high-density lipids, or statin use (p = 0.06).

Conclusion

The prevalence of microalbuminuria among T2DM patients is significantly high (39.1%) and is positively correlated with various factors such as male gender, hypertension, suboptimal control of diabetes mellitus, high HbA1c levels, longer disease duration, dyslipidemia with high triglycerides, treatment modalities of T2DM, and other diabetic complications like neuropathy and retinopathy. As diabetes is very prevalent in our country, the number of patients with diabetic kidney disease will rise significantly in the near future, leading to ESRD and other diabetic complications, and immediate intervention is needed to prevent this. Further research is warranted to explore potential interventions and evaluate their impact on patient outcomes.

## Introduction

The prevalence of type 2 diabetes mellitus (T2DM) is increasing worldwide and so are the disease-associated complications [1]. Approximately 540 million adults (one in 11) are living with diabetes worldwide; 20% of these develop diabetic nephropathy (DN), making it a global health concern [2]. The International Diabetes Federation (IDF) estimates that this prevalence of diabetes mellitus would reach 783 million (one in every eight adults) by 2045 [3].

T2DM is associated with microvascular issues like retinopathy, neuropathy, and nephropathy, as well as macrovascular complications such as myocardial infarction, stroke, and peripheral arterial disease. DN is a common and feared complication of diabetes mellitus, linked to higher morbidity and mortality rates [4]. It is also one of the leading causes of end-stage renal disease (ESRD) and the need for renal replacement therapy [5]. Both type-1 diabetes mellitus and T2DM can progress to ESRD, but T2DM is more common due to its higher prevalence [6].

Microalbuminuria (MA) refers to the presence of small amounts of albumin in the urine. It is a marker of kidney damage in both insulin-dependent and non-insulin-dependent diabetics, serving as an early indicator of nephropathy [7]. MA has been recognized as the "tip of the iceberg" when it comes to diabetic complications. It is an indicator of systemic endothelial dysfunction, which is characterized by impaired functioning of the inner lining of blood vessels. Endothelial dysfunction plays a crucial role in the development of atherosclerosis, a condition where fatty deposits accumulate in the arteries, leading to reduced blood flow and an increased risk of heart disease, stroke, and peripheral vascular disease [8]. This highlights the importance of early detection and intervention to mitigate the risk of developing these life-threatening complications.

The prevalence of MA in individuals with T2DM is significant. Studies have shown that approximately 20-40% of patients with T2DM develop MA within 10 years of diagnosis [9]. However, it is worth noting that MA can occur even in the absence of overt proteinuria and may be present in individuals with well-controlled blood glucose levels and concomitant hypertension [9,10].

Researchers have demonstrated that MA is a significant predictor of mortality and morbidity in patients with cardiovascular and peripheral vascular diseases, as well as an independent risk factor for ischemic heart disease in hypertensive and diabetic patients [10]. Early identification and management of MA can potentially reduce the risk of developing these complications, making it an essential aspect of comprehensive diabetes care. American Diabetes Association (ADA) has already included the screening of MA as a standard of medical care in diabetes [11].

The purpose of this study was to estimate the prevalence of MA and its relationship to hypertension and other diabetic complications among people with T2DM.

## Materials and methods

Study design

This descriptive, cross-sectional study was conducted at the outpatient diabetic clinic of Sheikh Zayed Medical College and Hospital, Rahim Yar Khan, Pakistan, from February 05, 2022, to February 10, 2023. The Ethical Research Review Board of Sheikh Zayed Medical College and Hospital approved the study (approval number: 230/IRB/SZMC/SZH). The inclusion criteria comprised patients of T2DM with a disease duration of more than five years, aged 35-60 years, and taking oral hypoglycemic drugs, insulin treatment or active lifestyle modifications. Patients with type 1 diabetes mellitus, newly diagnosed T2DM, secondary diabetes (medication-induced, endocrine disorder or hereditary disease), recent or current pregnancy, active infections, known chronic kidney disease or other associated comorbidities, and patients aged less than 35 years were excluded from the study.

Data collection

A total of 640 patients who met the inclusion criteria were selected. Patient interviews were conducted to gather information on their demographic and clinical characteristics, which included age, gender, duration of diabetes, treatment modalities, glycosylated haemoglobin (HbA1c), high-density lipoprotein (HDL), low-density lipoprotein (LDL), triglyceride levels, hypertension, dyslipidemia, diabetic retinopathy, peripheral neuropathy, and use of statins.

The participant's blood pressure was measured on their right arm while sitting using a standard sphygmomanometer. Patients were directed to provide a clean catch, mid-stream urine sample from their first morning void on the day following the visit, and were requested to report the urine albumin to creatinine ratio (ACR) (from the hospital laboratory). Meanwhile, other diabetic complications such as peripheral neuropathy were assessed using the monofilament and tuning fork test, while direct ophthalmoscopy was used to assess retinopathy. In addition, blood samples were sent to the laboratory for blood glucose, levels of HbA1C, and complete lipid profile (triglycerides, LDL, HDL).

Operational definitions

In accordance with major guidelines including American Heart Association, patients with blood pressure exceeding 140/90 mmHg or presently taking antihypertensive medications were designated as hypertensive. T2DM is characterized by hyperglycemia caused by insufficient insulin or resistance to insulin. The criteria for diagnosis include fasting glucose levels of ≥ 7.0 mmol/L and random plasma glucose levels of ≥ 11.1 mmol/L, self-reported disease and use of oral hypoglycemic drugs or insulin. MA is the presence of albumin levels ranging from 30 mg to 300 mg in a 24-hour urine collection or urinary ACR ≥ 3.5 mg/mmol (but < 30 mg/mmol) in females and ≥ 2.5 mg/mmol (but < 30 mg/mmol) in males. In contrast, macroalbuminuria is defined as urinary albumin excretion exceeding 300 mg in a 24-hour collection or urinary ACR more than 30 mg/mmol.

Data analysis

IBM SPSS Statistics for Windows, Version 21.0 (Released 2012; IBM Corp., Armonk, New York, United States). The demographic and clinical characteristics were matched between the groups with MA values of less than or more than 30 mg per day. The prevalence of hypertension between these groups was analyzed. To determine the frequency and percentage of these characteristics, descriptive analysis was performed. Means with SD were computed for statistical variables such as age and diabetes duration. The p-value <0.05 was considered significant for diabetic patients with MA. Adjusted odds ratio with 95% confidence intervals (CI) was compared for variable factors like hypertension, gender, HbA1c levels, and dyslipidemia.

## Results

**Figure 1:**
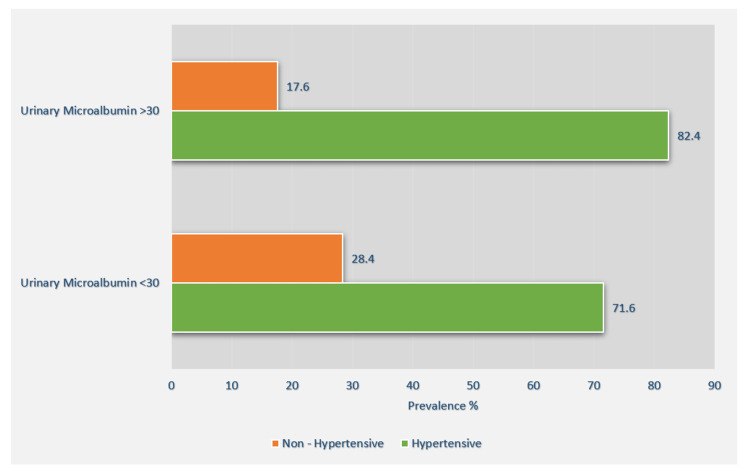
Prevalence of hypertension between patients with microalbuminuria <30 mg/day and >30 mg/day

A total of 640 patients were included in this study that was conducted from February 05, 2022, to February 10, 2023; all participants had T2DM. Of these, 47.1% (n=302) were male and 52.9% (n=338) were female. The mean age of the participants with MA was 53.9 with a SD of 6.1 years, and the mean duration of diabetes was 10.1 years with SD of 6.2 years**.** There were 485 (75.7%) T2DM patients with MA that were hypertensive. Urine ACR or MA >30 mg/day was found in 250 (39.1%) patients, 206 (82.4%) of whom had hypertension, while urine MA <30 mg/day was found in 390 (60.9%) participants of whom 279 (71.6%) had hypertension. The overall prevalence of MA in this study population was 39.1% (Figure 1).

HbA1c level was found to be 7.1-8 in 168 (26.3%) and ≥ 8.1 in 222 (34.7%). Dyslipidemia was diagnosed in 383 (59.9%), but 415 (64.8%) were using statins and 32 patients using statins empirically. In addition, 57.5% of the participants were on oral hypoglycemic drugs (OHD), 7.2 % on insulin, 32.9% on combination OHD and insulin, and 2.4% were on diet and lifestyle modification regimens. A comparison of the demographic and clinical characteristics of the study participants across the categories of urine MA levels is presented in Table 1.

**Table 1:** Demographic and clinical characteristics of participants (n=640) with microalbuminuria <30mg/day and >30mg/day Values are given as n (%) unless otherwise specified. SD: standard deviation; HbA1c: glycated haemoglobin; HDL: high density lipoprotein; LDL: low density lipoprotein; OHD: oral hypoglycaemic drugs

Characteristics	Overall (n=640)	Urine ACR or Microalbuminuria <30 mg/d (n=390)	Urine ACR or Microalbuminuria >30 mg/d (n=250)	p-value
*Age (Years*), mean ± SD	52.3 ± 7.7	49.2 ± 11.8	53.9 ± 6.1	0.56
Gender				
Male	302 (47.1)	201 (66.6)	101 (33.4)	0.05
Female	338 (52.9)	235 (69.5)	103 (30.5)	0.08
Hypertension				
Present	485 (75.7)	279 (71.6)	206 (82.4)	<0.001
Absent	155 (24.3)	111 (28.4)	44 (17.6)	<0.001
*Duration of Diabetes*, mean ± SD	9.6 ± 5.1	9.3 ± 4.8	10.1 ± 6.2	0.04
HbA1C%				
≤ 7.0	250 (39)	172 (44.1)	78 (31.4)	<0.002
7.1-8.0	168 (26.3)	104 (26.8)	64 (25.7)	<0.002
8.1 and above	222 (34.7)	114 (29.1)	108 (42.9)	<0.001
Dyslipidemia				
Present	383 (59.9)	219 (56.4)	164 (65.8)	0.001
Absent	257 (40.1)	171 (43.6)	86 (34.2)	0.08
LDL				
Normal (<100 mg/dl)	444 (69.4))	269 (69)	175 (72)	0.49
High (>100 mg/dl)	196 (30.6)	121 (31)	75 (28)	0.49
Triglycerides				
Normal (<150 mg/dl)	394 (61.6)	254 (65)	140 (56)	0.03
High (>150 mg/dl)	246 (38.4)	136 (35)	110 (44)	0.03
HDL				
Normal (≥ 50 mg/dl)	376 (58.7)	218 (56)	158 (63)	0.06
Low (≤ 49 mg/dl)	264 (41.3)	172 (44)	92 (37)	0.06
Diabetic Retinopathy				
Present	44 (6.9)	20 (5.1)	24 (9.7)	<0.002
Absent	596 (93.1)	370 (94.9)	226 (90.3)	<0.002
Peripheral Neuropathy				
Present	121 (18.9)	59 (15.1)	62 (24.6)	<0.001
Absent	519 (81.1)	331 (84.9)	188 (75.4)	<0.001
Statins Use				
Present	415 (64.8)	241 (61.8)	174 (69.6)	0.06
Absent	225 (35.2)	149 (38.2)	76 (30.4)	0.06
Treatment T2DM				
Insulin	46 (7.2)	27 (6.9)	19 (7.6)	<0.001
OHD	368 (57.5)	245 (62.8)	123 (49.2)	<0.001
Insulin + OHD	211 (32.9)	109 (27.9)	102 (40.8)	<0.001
Diet + Exercise	15 (2.4)	9 (2.4)	06 (2.4)	0.04

There was a statistically significant correlation between MA > 30 mg/d and hypertension, HBA1C level, diabetes mellitus control, dyslipidemia, therapy type, and history of diabetes retinopathy and peripheral neuropathy. However, there was no statistically significant correlation between MA and age, gender, diabetes duration since diagnosis, LDL, HDL, or statin use.

MA was more prevalent among patients with older age, male gender, longer duration of T2DM, prescribed OHD, poorly controlled T2DM with HbA1c levels above 8, dyslipidemia, elevated triglycerides, and a history of hypertension as opposed to patients who were younger, female, had diabetes for a shorter period of time, were on insulin, were well controlled, and did not suffer from hypertension or other microvascular complications associated with T2DM. Table 2 compares the variable factors associated with MA >30 mg/d in the 640 study participants.

**Table 2:** Factors associated with microalbuminuria >30mg/day Number of participants=640 HbA1C: glycosylated haemoglobin

Variable Factors	Unadjusted Odds Ratio (95% CI)	Adjusted Odds Ratio (95% CI)	p-value
*Hypertension*			
Yes	1.82 (1.35 - 2.45)	1.79 (1.31 - 2.44)	<0.001
No	1	1	0.06
*Gender*			
Male	1.36 (1.09 - 1.74)	1.42 (1.10 - 1.82)	0.05
Female	1	1	0.08
*HbA1C (%)*	1.20 (1.12 – 1.29)	1.23 (1.15 – 1.34)	<0.001
*Dyslipidemia*			
Yes	1.54 (1.21 – 1.98)	1.53 (1.18 – 1.98)	0.001
No	1	1	0.08

This study found that hypertension increased the odds of MA by 78% when adjusted for gender, HbA1c, and dyslipidemia (adjusted OR (aOR) 1.79, 95%CI: 1.31-2.44).

## Discussion

Several studies have been conducted to determine the prevalence of MA or urinary ACR in diabetes patients. MA leads to an increased risk of DN leading to ESRD or diabetic kidney disease. These studies provide valuable insights into the burden of this condition and help in identifying populations at high risk.

The prevalence of MA in this study was 39.1% and it’s comparable with the prospective diabetes study by Ufuoma et al. that showed the prevalence of nephropathy as 30.8% [12]. In Asian countries, the overall prevalence of MA is 20.3% in Nepal [13], 20-40% in China [14], 36.3% in India [15], 13% in Japan [16], and 14.3% in Iran [17] with contributing factors ranging from age, diabetes control HbA1c, blood pressure to lipid levels in these patients. The study by Kim et al. reported anaemia as a reason for the rapid decline of renal dysfunction and faster initiation of dialysis in DN patients [18]. The study by Asadujjaman et al. reported a prevalence of 29.72% MA in diabetic participants of Bangladesh [19], while Wu et al. reported a prevalence of 24.2% in Pakistan and 56.5% in Korea [20]. Variations in lifestyle, ethnicity, and education level may account for the huge difference in prevalence.

The study revealed a significantly higher male population in the microalbuminuric group, as has been observed in previous studies [12,13,18,19]. Research has shown longer duration and poor glycemic control of diabetes, male gender, and high creatinine as significant risk factors for microalbuminuria. These studies, however, were unable to demonstrate any difference between the two groups in terms of their age, gender, or levels of LDL and HDL, which should be investigated in further studies.

Besides being associated with ESRD in diabetics, MA is an important marker of mortality for diabetics [21]. The American Diabetics Association has emphasized the importance of early detection of MA in diabetes patients because early treatment retards DN progression [22]. The presence of hypertension and high HbA1c not only constitutes a risk factor, but it also correlates strongly with MA [23]. A study by Muddu et al. demonstrated that a significantly higher percentage of patients with MA had hypertension and uncontrolled diabetes with high HbA1c levels [24].

Mohammad et al. have demonstrated a direct correlation between raised HbA1c and MA [25]. Although age, serum creatinine, and hypertension have been identified as significant factors in MA in prior research, this study did not find a significant association between age and MA.

The results of the current study demonstrate a higher prevalence of chronic complications associated with diabetes, such as diabetic retinopathy and DN in patients with MA compared to those without. Existing literature indicates that timely intervention can mitigate these complications [26]. Patients with MA also showed a significantly higher incidence of dyslipidemia, particularly in triglyceride levels where HDL and LDL fail to show any significant difference [27]. Dyslipidemia represents a significant risk factor for macrovascular disease in individuals with diabetes, thus increasing their susceptibility to cardiovascular events [28]. The current study also draws attention to the greater utilization of statin therapy in patients with albuminuria, similar to other studies [26,28-30]; nonetheless, the efficacy of lipid-lowering therapy in reducing the risk of cardiovascular events in the Asian population remains unclear [30].

Additionally, analysis in the current study demonstrated a higher usage of insulin in individuals with MA as compared to those without, indicating the challenge of managing their diabetes through OHD alone.

The study was limited by patient exclusion criteria, which included: patients with type 1 diabetes mellitus, age less than 35 years, newly diagnosed diabetes, secondary diabetes, recent or current pregnancy, active infections, known chronic kidney disease, or other associated comorbidities.

## Conclusions

The prevalence of MA among T2DM patients is significantly high (39.1%) and is positively correlated with various factors such as male gender, hypertension, suboptimal control of T2DM, high HbA1c levels, longer disease duration, dyslipidemia with high triglycerides, treatment modalities of T2DM, and other diabetic complications, like neuropathy and retinopathy. As diabetes is very prevalent in Pakistan, the number of patients with diabetic kidney disease will rise significantly in the near future, leading to ESRD and other diabetic complications, and immediate intervention is needed to prevent this. Further research is warranted to explore potential interventions and evaluate their impact on patient outcomes.
